# German translation of the Alberta context tool and two measures of research use: methods, challenges and lessons learned

**DOI:** 10.1186/1472-6963-13-478

**Published:** 2013-11-16

**Authors:** Matthias Hoben, Cornelia Mahler, Marion Bär, Sarah Berger, Janet E Squires, Carole A Estabrooks, Johann Behrens

**Affiliations:** 1 Network Aging Research (NAR), Ruprecht-Karls-University Heidelberg, Bergheimer Str. 20, Heidelberg 69115, Germany; 2 Institute of Health and Nursing Sciences, Medical Faculty, Martin-Luther-University Halle-Wittenberg, Halle-Wittenberg, Germany; 3 Department of General Practice and Health Services Research, University Hospital, Ruprecht-Karls-University Heidelberg, Heidelberg, Germany; 4 Institute of Gerontology (IfG), Ruprecht-Karls-University Heidelberg, Heidelberg, Germany; 5 School of Nursing, Faculty of Health Sciences, University of Ottawa, Ottawa, Canada; 6 Clinical Epidemiology Program, Ottawa Hospital Research Institute, Ottawa, Canada; 7 Faculty of Nursing, University of Alberta, Edmonton, Canada

**Keywords:** Translation, Cultural adaptation, Alberta Context Tool, Estabrooks’ Kind of Research Utilization, Conceptual Research Use scale, Organizational context, Research utilization, Residential long term care

## Abstract

**Background:**

Understanding the relationship between organizational context and research utilization is key to reducing the research-practice gap in health care. This is particularly true in the residential long term care (LTC) setting where relatively little work has examined the influence of context on research implementation. Reliable, valid measures and tools are a prerequisite for studying organizational context and research utilization. Few such tools exist in German. We thus translated three such tools (the Alberta Context Tool and two measures of research use) into German for use in German residential LTC. We point out challenges and strategies for their solution unique to German residential LTC, and demonstrate how resolving specific challenges in the translation of the health care aide instrument version streamlined the translation process of versions for registered nurses, allied health providers, practice specialists, and managers.

**Methods:**

Our translation methods were based on best practices and included two independent forward translations, reconciliation of the forward translations, expert panel discussions, two independent back translations, reconciliation of the back translations, back translation review, and cognitive debriefing.

**Results:**

We categorized the challenges in this translation process into seven categories: (1) differing professional education of Canadian and German care providers, (2) risk that German translations would become grammatically complex, (3) wordings at risk of being misunderstood, (4) phrases/idioms non-existent in German, (5) lack of corresponding German words, (6) limited comprehensibility of corresponding German words, and (7) target persons’ unfamiliarity with activities detailed in survey items. Examples of each challenge are described with strategies that we used to manage the challenge.

**Conclusion:**

Translating an existing instrument is complex and time-consuming, but a rigorous approach is necessary to obtain instrument equivalence. Essential components were (1) involvement of and co-operation with the instrument developers and (2) expert panel discussions, including both target group and content experts. Equivalent translated instruments help researchers from different cultures to find a common language and undertake comparative research. As acceptable psychometric properties are a prerequisite for that, we are currently carrying out a study with that focus.

## Background

Gaps between research-informed best practice and actual health care practice exist across countries, health care disciplines, and settings [1-5]. Many people thus receive care that is less effective, ineffective or potentially harmful [5]. Closing the research-practice gap is complex and challenging [6-15]. Myriad influencing factors at interacting levels (e.g., structural, organizational, innovation, health care provider, care recipient characteristics) contribute to this complexity [16]. Characteristics of the organizational context are believed to be particularly important for research implementation [12,15-18]. Contextual factors such as feedback mechanisms, available information resources, etc. are potentially modifiable. They influence research utilization by individuals and teams and can be used effectively to improve research implementation [19-25].

Research implementation is under-investigated in the residential long term care (LTC) setting [26-29]. LTC is a complex care environment for highly vulnerable residents with cognitive and physical impairments; residents are threatened if health care staff provide less than best practices [30-32]. Current international research programs e.g., [28,33-35] propose to increase our understanding of organizational context in LTC, how it relates to research implementation and care quality, and how both can be improved. German LTC settings show substantial evidence of suboptimal use of best practices and resulting poor quality e.g. [32,36-41]. We thus intended to study the influence of organizational context on research implementation in German residential LTC.

Reliable and valid assessment instruments are essential for such a study [16,42]. We sought a tool that (1) was constructed on a sound theory and evidence base, (2) assessed potentially modifiable organizational context factors thought to influence research utilization, (3) was specifically adapted for use with various provider groups, (4) was available in a version for residential LTC settings, (5) was brief, and (6) had acceptable psychometric properties. Chaudoir et al. [16] identified 62 measures to assess factors affecting the implementation of health innovations; the Alberta Context Tool (ACT) [43-47] best met our requirements. No available German measures matched our optimal characteristics. Sarges and Wottawa [48] listed 40 German organization psychological assessment instruments; only one is designed for use in healthcare settings – the *Tätigkeits- und Arbeitsanalyseverfahren* (TAA or Task and Job Analysis Tool, available in a residential LTC version) [49]. The TAA, like other German health care specific context assessment instruments (e.g., the revised Nursing Work Index [50,51]), is not designed to assess context from the perspective of research utilization. Therefore, we chose to translate the ACT into German. No German instruments are available to assess research utilization, thus we included two additional instruments: Estabrooks’ Kinds of Research Utilization (RU) measure [52-54] (residential LTC version [21]) and the Conceptual Research Use (CRU) Scale, developed by Squires et al. [55].

The ACT was developed for adult acute care and then adapted for pediatric acute care [44,45], residential LTC [47], and home care [46]. Specific forms are available for six provider groups: (1) healthcare aides (HCAs), (2) registered nurses (RNs), (3) physicians, (4) allied health professionals (AHPs), (5) practice specialists, and (6) care managers [23]. The forms differ slightly in number of items (56 to 58), structure of item stems and examples of concepts [23]. The ACT contains 10 organizational context concepts based on the Promoting Action on Research Implementation in Health Services (PARIHS) framework [18,56] and related literature [57,58]: (1) leadership, (2) culture, (3) evaluation, (4) social capital, (5) informal interactions, (6) formal interactions, (7) structural and electronic resources, (8) organizational slack (staff), (9) organizational slack (space), and (10) organizational slack (time) [46]. Initial psychometric assessments of pediatric acute care nurse responses [44] provided evidence for acceptability, internal consistency reliability (α ≥ 0.70 for 10 of 13 concepts), and validity: Principal component analysis suggested a 13-factor solution, statistically significant correlations between instrumental RU and all but one ACT concepts were found. Psychometric properties of the residential LTC version were assessed based on HCA responses [23]: The Overall data pattern, evaluated by three different confirmatory factor models, was consistent with the hypothesized 10-factor structure. For eight ACT concepts, significant correlations were reported with instrumental RU; internal consistency reliability (α ≥ 0.70 for 8 of 10 concepts), and acceptability were confirmed.

Estabrooks’ Kinds of RU measure assesses research use as (1) instrumental, (2) conceptual, (3) persuasive and (4) overall. Each use is introduced by a definition and examples and then assessed by a single item, asking participants how often they used research that way on their last typical day of work. Squires et al. [59] identified 10 articles assessing psychometric properties, providing evidence for content and response process validity. Significant relations to other variables were demonstrated, especially attitudes towards research use.

The CRU Scale contains five items asking participants how often best practice knowledge accomplished something e.g., giving new knowledge or changing their mind. A psychometric assessment with HCAs [55] provided evidence for acceptability, reliability, content and response process validity. The authors report significant associations of CRU items with other RU concepts and belief suspension. Results for internal structure validity were inconsistent; the 5-item 1-factor model suggested by principal component analysis was not supported by confirmatory factor analysis. Best fit was attained with a 4-item 1-factor model.

Translating an assessment instrument for use in a different culture requires rigorous methods to ensure equivalence of the original and translated versions [60-62]. Cross-cultural comparison of results necessitates conceptual, semantic, operational and psychometric measurement equivalence^a^[60,63]. Conceptual equivalence is achieved when the number and definitions of constructs are the same in both instruments and users of both cultures know all constructs, rate them as relevant and accept them. Semantic equivalence is achieved when items have the same meaning for users of both cultures. Operational equivalence requires the same survey administration methods in both cultures: mode of administration, questionnaire format, reading level, instructions, item format and respondent burden. Psychometric measurement equivalence is achieved when psychometric quality of both instruments is comparable and acceptably high [60,63]. Guidelines suggest best practices of instrument translation to ensure equivalence of source and target versions, but guidelines differ in many aspects (e.g., definitions of “equivalence”, “culture” or “adaptation”; kind, design and order of process steps; persons involved; reporting requirements) with no “gold standard” [60,62,64,65]. Translators must therefore make their steps transparent and justify the design of the translation process. Detailed documentation of the process (1) enables translators, reviewers and instrument users to trace back difficulties and solutions, (2) facilitates interpretation of psychometric testing results and instrument scores, and (3) shows benefits and limits of the translated instrument [60,66].

Few reports are published yet, but several groups have translated the ACT and use it in their cultural contexts. It has been translated into Dutch, Swedish, Mandarin Chinese and French, and used in studies in eight countries: Canada, United States, Sweden, Netherlands, United Kingdom, Republic of Ireland, Australia and China [46]. In a recent publication, Eldh et al. [67] report on translation of the ACT residential LTC RN form, highlighting challenges, solutions and preliminary results for validity, acceptability and reliability. Experiences and specific challenges in translating ACT forms for other provider groups, and the two RU tools, have not been reported. The German LTC setting and context differ from other countries, with unique challenges and adaptation needs. This article describes the translation process, providing a rationale for the translation methods chosen and the strategies applied to challenges. We point out challenges and strategies for their solution unique to German residential LTC, and demonstrate how resolving specific challenges in translating the HCA instrument versions facilitated our translation of the tools for the other providers (RNs, AHPs, specialists, and managers). We aim to facilitate further translations of the ACT and RU measures or similar tools designed for residential LTC providers.

## Methods

### Overall project design

The project of translating and validating the three tools was divided into three major phases, each with different methods and samples. The first phase was the translation process, which we report here. In the second phase, we conducted a linguistic validation of the translated tools, based on responses from 39 participants (16 HCAs, 5 RNs, 7 AHPs, 5 specialists and 6 managers) from five German LTC facilities, to be reported elsewhere. In phase 3 we are conducting the formal validation of the translated tools in a second sample of 821 care providers (273 HCAs, 196 RNs, 152 AHPs, 6 specialists, 129 managers and 65 nursing students) from 38 German LTC facilities using confirmatory factor analyses. We will also investigate the associations between the individual provider level variables (e.g., attitudes toward research use, job satisfaction, stress, etc.), the ACT variables, and the RU scores using regression and structural equation modeling.

### Translation process design

The translation process followed the principles of good practice for translation and adaptation from the Translation and Cultural Adaptation work group of the International Society of Pharmacoeconomics and Outcomes Research (ISPOR) [62]. Based on McKenna and Doward’s [68] critical discussion, we added an expert panel step. McKenna and Doward [68] argue that the back translation step might be insufficient to ensure the equivalence of the source and target instrument. In their own two-panel approach, forward translations are produced by a panel of eight to twelve professionals, then discussed and adapted within a panel of two to eight lay people; no back translation is created [69,70]. This helps ensure “quality in the translation, in addition to checking it a posteriori” [68] (p.89). In contrast, we kept the back translation step to promote close cooperation with the tool developers and acquaint them with the German version. We thus combined the independent forward and back translation method with a target group expert panel. For methodological rigor, we used a checklist developed by Acquadro et al. [60] to design, monitor and document the translation process.

**Figure 1:**
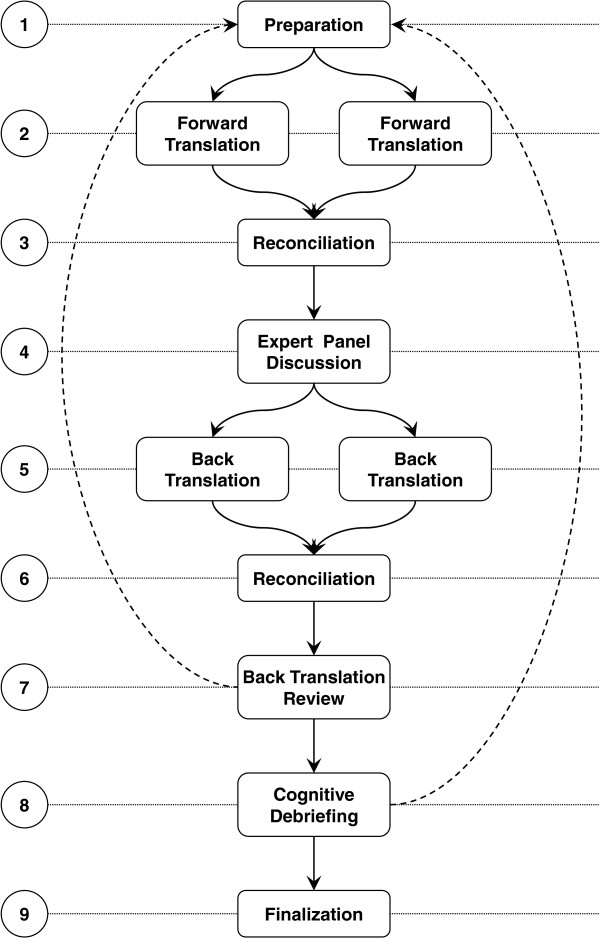
Steps in the ACT and research utilization tools translation process.

We translated five of the six ACT forms (HCA, RN, AHP, specialist, manager), beginning with the HCA tools (Figure 1). After the full translation process for all HCA tools, we translated the RN tools. Wordings that were the same in the RN tools and approved in the HCA tools were adopted directly; only differing wordings required translation. AHP, specialist and manager forms were then translated sequentially.

### Step 1: Preparing the translation process

We developed a proposal and timeline for the translation process, seeking and receiving approval for this work from the tool developers. They agreed to work with us on the translation, providing us with original questionnaires and concept maps defining each construct in detail. Next, we recruited forward and back translators and expert panel members. Finally, we developed standardized template forms for forward and back translations and expert panel discussions.

### Step 2: Forward translation

Following the ISPOR guideline [62], two persons (MH and MB) independently forward translated the instruments. Each instrument contained multiple elements to translate: (1) lead texts introducing the instrument, (2) stem texts introducing concepts and guiding participants in handling items, (3) item texts, (4) skip patterns determining which item is followed, based on coding of the previous item, and (5) texts of item rating scales. Both translators are native German speakers with excellent English skills. They are familiar with provider groups, instrument concepts and contextual conditions in residential LTC through clinical experience in nursing practice and LTC research experience (MB is a gerontologist, MH is a nursing science researcher). Questions arising during the translation process, such as construct meanings, wordings used or the background of instrument development, were discussed with the developers. The translators did not discuss their forward translations with each other until both versions were fully completed.

### Step 3: Reconciliation of the forward translation

The two forward translators discussed each text element according to three criteria: (1) whether they found it easy or difficult to translate, (2) whether the two independent translations had no, slight or strong discrepancies, and (3) whether a reconciled version was easy or difficult to find. An element was rated difficult to translate if the translators spent a long time translating it, had difficulty finding appropriate wording, tried out many different translations and found it hard to opt for one. Discrepancies were rated as slight if the meaning was almost the same but somewhat different grammar or synonyms were used. Discrepancies were rated as strong if the meaning differed significantly. Segments matching exactly, differing slightly or differing strongly were found in both elements that were easy to translate and elements that caused difficulties. Table 1 provides an example of each of these combinations.

**Table 1:** Examples of items that were easy or difficult to translate with no, slight or strong discrepancies of the two forward translations

	**Easy to translate**	**Difficult to translate**
**Translations matched exactly**	**ACT: Time, item 1**	**ACT: Feedback, stem**
	**Original wording:** How often do you have time to do something extra for residents? **Translations 1 and 2:** Wie oft haben Sie Zeit, um für Bewohner auch mal etwas zusätzlich zu tun?	**Original wording:** Sometimes you may receive formal information about the care that is being provided to residents.
		**Translations 1 and 2:** Es kann sein, dass Sie von Zeit zu Zeit formale, also offizielle, Informationen über die von Ihnen geleistete Pflege erhalten.
**Translations differed slightly**	**ACT: Slack – space, item 1**	**ACT: Leadership, item 5**
	**Original wording:** We have adequate space to provide resident care.	**Original wording:** The leader actively mentors or coaches performance of others.
	**Translation 1:** Wir verfügen über genügend Platz, um die Versorgung der Bewohner zu gewährleisten.	**Translation 1:** Die Führungsperson betreut oder berät die Mitarbeiter hinsichtlich Ihrer Leistung.
	**Translation 2:** Wir haben für die Pflege der Bewohner genügend Räumlichkeiten zur Verfügung.	**Translation 2:** Die Führungsperson berät und betreut aktiv die Arbeitsleistungen anderer.
**Translations differed strongly**	**ACT: Connections among people, item 4**	**Research utilization: instrumental research use, item 1**
	**Original wording:** I am comfortable talking about resident care issues with those in positions of authority.	**Original wording:** On your last typical work day, how often did you use this type of best practice knowledge to provide resident care?
	**Translation 1:** Ich fühle mich wohl dabei, mit Personen in verantwortlichen Positionen über die Pflege und Betreuung der Bewohner zu sprechen.	**Translation 1:** Wenn Sie an Ihren letzten typischen Arbeitstag denken: Wie häufig haben Sie diese Art Wissen zur optimalen Praxisgestaltung bei der Pflege und Betreuung von Bewohnern angewendet?
	**Translation 2:** Ich kann mit höher qualifizierten Mitarbeitern gut über Themen, die die Pflege der Bewohner betreffen, sprechen.	**Translation 2:** Wenn Sie an Ihren letzten typischen Arbeitstag denken: Wie oft haben Sie solche neuartigen Erkenntnisse, Instrumente oder Konzepte in der Pflege der Bewohner eingesetzt?

The translators discussed discrepancies until consensus was reached on which translation was closer to the original wording, would be better understood by the intended audience, used better grammar and wording, and was shorter. Reconciliation results and justification of decisions were documented.

### Step 4: Expert panel discussion

Expert panel discussions ensured cultural adaptation, content validity and comprehensibility of translated instruments. The expert panel discussed each element rated as difficult to translate, difficult to reconcile, or for which the translations differed substantially. Other elements were discussed if requested by a translator or an expert panel member. Expert panel discussions were necessary only for the HCA and RN form translations; almost all wording of the HCA and RN forms could be adopted for the AHP, specialist and manager forms. Remaining differences were minimal (e.g., slightly differing examples or focus on facility rather than care unit). The four members of the HCA expert panel include one nursing science researcher with expertise in elder care and residential LTC, one university lecturer for future elder care educators, one elder care educator and one RN working in residential LTC (Table 2). In their various roles, each of the experts deals extensively with HCAs.

**Table 2:** Expertise of the HCA expert panel discussion members

	**Current job/role**	**Qualifications**	**Job experience**
**Expert 1**	Nursing science researcher with a particular expertise in elder care and residential LTC	RN	7 years as RN in residential LTC
		University diploma in nursing	7 years as nursing science researcher
**Expert 2**	University lecturer in a program educating future elder care educators	RN	6 years as RN in various settings
		University diploma in nursing education	23 years as elder care educator
			5 years as university lecturer and nursing science researcher with a particular expertise in elder care and residential LTC
**Expert 3**	Elder care educator	RN	6 years as RN in various settings
		University diploma in nursing education	2 years as university lecturer in a program educating future elder care educators
			8 years as elder care educator
**Expert 4**	RN in residential LTC	RN	10 years as RN in residential LTC

The members of the RN expert panel have similar backgrounds to the members of the HCA panel (Table 3). Expert 1 participated in both panels.

**Table 3:** Expertise of the RN expert panel discussion members

	**Current job/role**	**Qualifications**	**Job experience**
**Expert 1**	Nursing science researcher with a particular expertise in elder care and residential LTC	RN	7 years as RN in residential LTC
		University diploma in nursing	7 years as nursing science researcher
**Expert 5**	Elder care educator	RN	15 years as RN in various settings
		University diploma in nursing education	21 years as elder care educator
**Expert 6**	RN in residential LTC	RN	5 years as RN in residential LTC

One week before each expert panel discussion, members received a template with English wording for each element, the corresponding German translation, and comments including specific questions from the translators or whether the element was an optional or obligatory discussion point (example template, Table 4).

**Table 4:** Example section of the template for expert panel discussion members

**Original Wording**	**Consented forward translation**	**Comments**
My organization effectively balances best practice and productivity	Meine Organisation schafft erfolgreich die Balance zwischen optimaler Versorgungsqualität und wirtschaftlicher Produktivität	Item needs to be discussed in the expert panel
		Particular focus: German translation of “best practice” and “productivity”

Panelists were asked to critically review whether the translation (1) correctly reflected the English meaning, (2) was relevant for the audience, HCAs or RNs in German residential LTC, and (3) was understandable by care providers. The two forward translators attended expert panel discussions but took no active part in discussing items; they answered questions, provided background information and documented discussion results. One translator (MB) moderated discussions. Unsettled questions were considered afterwards with the tool developers.

### Step 5: Back translation

The German version was independently translated back into English by two persons (CM and SB) familiar with residential LTC and provider groups. Both are experienced nurses and nursing science researchers. One (SB) is a native English speaker, the other (CM) has spoken English since early childhood. Neither had previous exposure to the original instruments but they were familiar with health care concepts assessed by the tools. Neither was involved in steps 2 through 4. The translators did not discuss their back translations with each other until both versions were fully completed.

### Step 6: Reconciliation of the back translations

As in step 3, the two back translators compared their versions and discussed discrepancies. Decisions and their justifications were documented.

### Step 7: Back translation review

The instrument developers compared the reconciled back translation to their original version, evaluating if wording changes altered the meaning and intent of each original element. If necessary, the German forward translations for specific element wordings were modified and new back translations created. This cycle was repeated until all element translations were accepted by the developers.

### Step 8: Cognitive debriefing

Methods and results for this step are described in detail in a second publication, thus they are outlined here only briefly. Cognitive debriefing includes a linguistic validation procedure to “assess the clarity, intelligibility, appropriateness, and cultural relevance of the target language version to the target population” [71] (p. 47). It evaluates whether participants understand the meaning of questionnaire elements as intended by the instrument developers [60,62,72,73]. We used a qualitative, semi-structured cognitive interviewing method called *verbal probing*[74]. Thirty-nine participants (16 HCAs, 5 RNs, 7 AHPs, 5 specialists, 6 managers) from six nursing homes completed the ACT and RU questionnaires and then were asked to explain their answers in detail for selected items. Interviews were recorded, transcribed and paraphrased. Meanings described by participants were compared to concept maps created by the instrument developers to define each construct in detail. If at least two participants gave non-matching answers for an item, the translation team discussed whether revision was required. Modified items were back translated into English and reviewed by the tool developers, followed by a new cognitive debriefing with new target group participants. The HCA translation required three cognitive debriefing rounds, but other translations required only one. Debriefing rounds ended when all wordings were understood by target persons.

### Step 9: Finalization

The translation was considered final with developers’ approval after step 8.

### Identification and categorization of challenges

**Figure 2:**
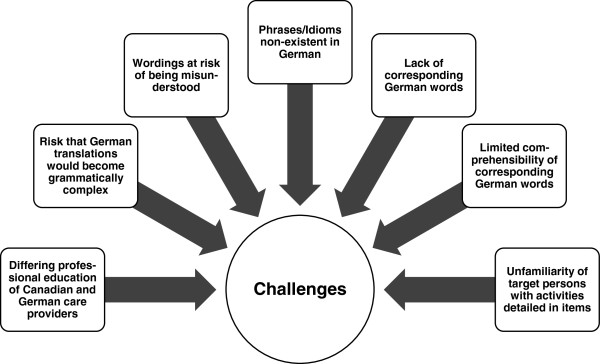
Challenges occurring during the translation process.

We documented each step of the translation process in detail with regard to methods used, participants involved, results, challenges met, and decisions made. Based on these protocols and on the experiences of the translation team members, we discussed the challenges that occurred during the translation process and clustered them thematically into seven categories (Figure 2).

## Results

**Figure 3:**
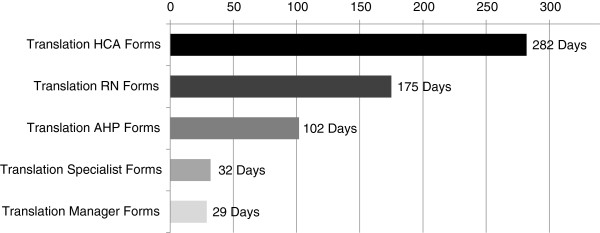
Time needed for the translation of the five sets of forms.

The complete translation process took 16 months, beginning with translation of the HCA forms and ending with proofreading and formatting of all questionnaires. Translation of the HCA forms took the longest, 286 calendar days (i.e., time that passed between the start of the translation and final approval of the translated versions by the tool developers, including waiting times, weekends, holidays, etc.), with the time needed for translation decreasing with each subsequent set of forms: 175 days RN, 102 days AHP, 32 days specialist, and 29 days manager (Figure 3). Reasons included:

1) The number of elements to be translated decreased with each set of forms. The HCA form had to be translated completely but wordings that matched and were already approved in previous forms could be adopted directly.

2) Translation of the HCA forms offered the most challenges in finding appropriate wordings. This provider group has the lowest education level and most heterogeneous language skills.

3) The translators learned during the translation process. Their skills in finding the right wording, anticipating problems and finding appropriate solutions increased with each set of forms.

### Challenges

Challenges in the translation process (Figure 2) arose from a tension between two somewhat contradictory requirements. On the one hand, we needed to maintain quality and comparability with the Canadian original, staying as close as possible in number and meaning of concepts, items and scales. On the other hand, we wanted instruments that are reliable and valid in the new German context. We critically examined relevance and meaning of concepts, items and scales, taking into account structural and cultural differences between Canada and Germany.

Dealing with all these challenges and finding the balance between closeness to the Canadian original and cultural adaptation needs was especially relevant in translating the first set of forms, for the HCAs. Approval of all HCA items for cognitive debriefing required three rounds of back translation review (step 7). The developers requested revisions when a back translation did not match the original meaning sufficiently. We then discussed sources of differences: (1) an imprecise forward translation (requiring improvement), (2) necessary cultural adaptations of the forward translation (not requiring modification) or (3) back translation based on correct forward translation but using wordings differing strongly from the Canadian original (forward translation not requiring modification). The amount of time and effort dedicated to ensuring a robust translation process for the first tool (HCA) meant that, when it came to translating the other versions, process gains and streamlining were significant for each subsequent translation. For example, the RN translation (second form translated) required only two rounds of back translation review and the remaining forms only one round.

The cognitive debriefing (step 8) for the HCA translation again required three rounds, each followed by modification of the forward translation until all items were understood correctly. Modified wording was then reconciled in two rounds with the developers. All other forms underwent one cognitive debriefing round and were not further modified, thus not requiring reconciliation with the developers.

### Differing professional education of Canadian and German care providers

Although university education for nurses is offered in Germany, it is not required for professional qualification. Most RNs in German residential LTC facilities have completed vocational training, which alternates practice phases in care institutions with theory phases in federally regulated vocational schools (similar to regulated AHPs in Germany). This affected the RN and AHP translations in two ways. First, we could not assume that RNs or AHPs were as familiar as Canadian regulated providers with concepts like best practice, research and scientific knowledge. We thus stayed closer to the HCA wording than the Canadian original. Second, no equivalent to the Canadian “Licensed Practical Nurse” exists in Germany, thus in the German version we removed one ACT item referring to this group.

### Risk that German translations would become grammatically complex

German sentences are, for various reasons, often longer and more complex than English sentences with the same meaning. Reasons include, for instance, long German words; different gender forms of words; complex grammatical structure of German sentences with numerous relative clauses; English adjectives or verbs, which cannot expressed with a single German word, but require a sub-clause; etc. Therefore, translation of English to German is a challenging process. One of our English original items says:

“We have *private space* such as a conference room on this unit or floor (*other than* at the bedside, in the hallway or medication room) to discuss resident care plans and share knowledge about resident care and best practices.”

For example, the German translation of “private space” (*nicht öffentlicher Personal- oder Besprechungsraum*) is almost five times longer than the English phrase, and while there is one word in English for both, male and female residents, in German we use *Bewohner* (masculine) and *Bewohnerinnen* (feminine). When we initially tried to use only one German sentence to translate the Canadian wording, this text became 437 characters (including blanks) long (compared to 223 characters of the English sentence), and it was complex and difficult to read. Whenever a German version became too complex, we made two or more sentences out of one. We also decided to use only the male version of words with different gender forms, which is a common practice in German to keep sentences concise. This way we could substantially shorten our final translation of the above mentioned example item (262 characters) and increase its comprehensibility.

### Wordings at risk of being misunderstood

A literal translation into German of some original wording risked being misunderstood e.g., how often participants did something “in the last year”. If they were asked, for example, in December 2012 they might respond for occurrences in 2011 rather than occurrences from December 2011 to December 2012. We thus changed wording to “in the last 12 months”.

### Phrases/idioms non-existent in German

Phrases and idioms are shaped and characterized by the culture of the source country. Their pithy meaning results from their familiarity, an important element potentially lost in translation. We therefore faced a difficult challenge when looking for German equivalents. For example, one item asks participants to what extent they agree with the statement that there is enough staff to ensure “that residents have the *best day*”. A literal translation makes no sense in German and describing the meaning of “having the *best day*” destroys the pithiness of the phrase. Our German wording emphasizes the residents’ “*best possible wellbeing*” in their everyday life.

### Lack of corresponding German words

Some key original English terms have no matching appropriate German word e.g., “*to coach*”, “*best practice*” and “*performance*”. The term “*best practice*” was especially difficult to translate. We described the meaning (“knowledge of how to provide the best possible care quality”) in the German translation.

### Limited comprehensibility of corresponding German words

Although a clear and correct German translation exists for terms like “formal”, “Informal” or “rating scale” (formal = *formell*, informal = *informell*, rating scale = *Einschätzungsskala*), the direct translation would not be understood by many HCAs. Our strategy was to describe the term and add illustrative examples not included in the Canadian original.

### Target persons’ unfamiliarity with activities detailed in survey items

One survey item asks how often a provider participated in “family conferences” in the last typical month. Family conferences are common in Canadian LTC among the resident, family members and staff representatives (nurses, therapists, HCAs, social workers, nursing directors, etc.). In German nursing homes, exchanges with a resident’s family members are mostly unplanned. RNs and HCAs exchange information informally with relatives or respond to questions or requests. Facility instructors, nursing directors or some AHPs might organize planned meetings with family members but meetings including multiple provider groups are rare. Our challenge was to have providers think of the correct concept even if it was unfamiliar, and answer “never” or “rarely” if they participated only in their usual informal conversations. We therefore added the term “planned”.

Another survey item asks to what extent the participants agree that they “routinely receive information on their teams’ performance” based on a particular type of data, e.g. “*number of resident falls or pain control*”. In Canada residents are routinely assessed with the standardized Resident Assessment Instrument (RAI), allowing quality indicators to be derived systematically. Canadian facilities are then required to discuss indicators with staff. German LTC facilities are required to assess residents’ situations comprehensively, but no instruments are mandated. Each facility uses its own assessment forms and procedures, many of them self-developed or with questionable validity. German nursing homes must provide “risk management tables” recording the number of residents who e.g., fall or develop pressure ulcers. Feedback is normally on individual resident situations, not indicators for the whole unit or facility. We wanted providers to think of the correct concept and answer “strongly disagree” or “disagree” if they only received feedback on individual residents, not on indicators for multiple residents of a unit or facility. We therefore used the word “statistics” to underline this meaning, provided examples and emphasized in the item stem that individual resident information is not meant here.

## Discussion

In studies using translated instruments, comprehensive information about the translation process is critical for reviewers and readers to assess the adequacy of the translated instrument – however, it is rarely provided in sufficient detail [60,66]. In this article, we provide detailed insight into methods used, challenges arising and strategies chosen to meet challenges during translation of the ACT and the RU tools into German. Our study will facilitate translations of these instruments into other languages and German translations of similar tools. Our findings will support interpretation of psychometric testing results for the translated instruments (currently underway), contributing to understanding the instrument scores of any further studies using the German versions.

We were able to adapt the translated instruments to the context of German residential LTC and obtain equivalent Canadian and German questionnaires. Balancing these two requirements, we followed a rigorous, iterative translation process that was methodologically demanding and time-consuming [60,64]. Our challenges are comparable to those described in other studies on translation and cultural adaptation of questionnaires [67,75-78]. Our primary challenge was finding appropriate wordings for the HCA provider group. HCAs are a highly heterogeneous and mostly less-educated group in German residential LTC. Terms like “best practice” or “rating scale” were unfamiliar to them and they found it difficult to imagine the relevance of research knowledge to their work. In some cases no appropriate German translation was available (e.g., best practice), in others the German translation was not understood by the HCAs (e.g., rating scale). We thus struggled to find translations that were (1) understood by the HCAs, (2) did not alter the meaning of the original wording and (3) were concise. As with the Swedish ACT translation [67], discussions with the instrument developers were essential to understand the original concepts, avoid pitfalls and ensure equivalence. Expert panel discussions were vital to meeting adaptation needs and finding comprehensible wordings. Although several discussion, cognitive debriefing and reconciliation rounds were required in translating the HCA forms, the benefit became evident in translating the other provider group forms. Adopted wordings were understood without difficulty and the necessary number of rounds decreased with every provider group.

Available guidelines for translation processes are limited in only referring to translation of patient-reported outcomes. We found no best practice guideline for translation of staff-reported outcomes in health care or residential LTC. Squires et al. [61] have since developed a robust procedure for multi-language translation and harmonization of health services research instruments. Health care providers function in professional and organizational contexts that form subcultures in their country’s overall cultural context. Guidelines for translation of patient-reported outcomes do not focus on the consequences of these contexts for translation processes. Professional education and roles of provider groups may differ between the source and target countries: some provider groups might not exist in one country, professional terms might be used differently, etc. Squires et al. [61] (p. 265) note that “subtle differences in the conceptual meaning of words can often create completely different survey question structures and alter language use”. Translation process personnel must be familiar with instrument concepts as well as professional and cultural context of both source and target countries. While guidelines for translating patient-reported outcomes are a valuable resource in designing translation processes for staff-reported outcomes, specific guidelines like Squires et al. [61] will better meet the requirements of future translation for staff-reported outcomes instruments.

A rigorous process is necessary but not sufficient to ensure a comparable, reliable and valid translation. The translated instrument requires further assessment with both statistical methods and qualitative methods like cognitive interviewing [64,68,73,74]. In our study, we assessed the response process validity with cognitive debriefing (step 8) (to be reported elsewhere). We are currently testing psychometric properties of our translated instruments.

## Conclusions

Translating an existing instrument is inevitably complex and time-consuming to obtain cultural equivalence. Integrated involvement of and co-operation with the instrument developers is crucial. Adding expert panel discussions (steps 4 and 7) to the forward and back translation process improved the quality of our forward translations discernibly. Specific expertise of both questionnaire target groups and content experts was needed to overcome challenges during the translation process.

Cross-cultural comparison of research results notably improves our knowledge of organizational context and its relationship to research utilization. For valid cross-cultural comparison and interpretation of results, the translation process and its challenges must be rigorously documented. Thus the work in translating the ACT and the RU tools was worthwhile. Culturally equivalent translated instruments help researchers of different cultures to find a common language. Acceptable psychometric properties are also required; our translated instruments are currently being evaluated for these.

## Endnote

^a^Item equivalence and criterion equivalence are two subcategories of psychometric equivalence [63].

## Abbreviations

ACT: Alberta Context Tool; AHP: Allied Health Professional; CD: Cognitive debriefing; HCA: Health care aide; ISPOR: International Society of Pharmacoeconomics and Outcomes Research; LTC: Long term care; RN: Registered Nurse; RU: Research utilization.

## Competing interests

The authors declare that they have no competing interests.

## Authors’ contributions

MH led the development of the translation process design, led the research project, carried out the first forward translation and cognitive debriefings, and drafted this manuscript. CM assisted with the development of the translation process design and performed the first back translation. MB carried out the second forward translation, moderated the expert focus groups and contributed to the analysis of the cognitive debriefings. SB did the second back translation. CAE and JES developed the ACT and the two RU measures, approved the translation of the tools, did the back translation reviews and provided advice and recommendations regarding the translation process design and the translations. JB supervised the entire project. All authors contributed to the research design and contributed to, read, and approved the final version of this manuscript.

## Pre-publication history

The pre-publication history for this paper can be accessed here:

http://www.biomedcentral.com/1472-6963/13/478/prepub
